# Data for quantum phonon transport in strained carbon atomic chains bridging graphene and graphene nanoribbon electrodes

**DOI:** 10.1016/j.dib.2018.11.049

**Published:** 2018-11-15

**Authors:** Hu Sung Kim, Tae Hyung Kim, Yong-Hoon Kim

**Affiliations:** School of Electrical Engineering and Graduate School of EEWS, Korea Advanced Institute of Science and Technology (KAIST), 291 Daehak-ro, Yuseong-gu, Daejeon 305-701, Republic of Korea

## Abstract

The data presented in this article are related to “Odd-even phonon transport effects in strained carbon atomic chains bridging graphene nanoribbon electrodes” (Kim et al., 2019). To provide the information that can be utilized in reproducing our results, we provide the fully optimized graphene nanoribbon (GNR)–carbon chain (CC)–GNR junction atomic configurations at different strain values, the computational setting for quantum phonon transport calculations, and several computational data that were not included in the main manuscript. Data on graphene–CC–graphene junctions are additionally presented.

**Specifications table**

**Table:** 

Subject area	Physics
More specific subject area	Computational condensed matter
Type of data	Table, figure, and file
How data were acquired	Computational modeling and simulations
Data format	Analyzed
Experimental factors	1) Electrodes: hydrogen-passivated four zigzag-chain zigzag GNR (4zGNR), hydrogen-passivated seven dimer-line armchair graphene nanoribbon (7aGNR), and zigzag-edged graphene (aGRP) 2) Channels: 5–10 CCs for the 4zGNR case, 3–6 CCs for the 7aGNR case, and 5–6 CCs for the aGRP case 3) Tensile strains: imposed by extending the left and right GNR or GRP electrodes outward by the 0.4 Å step size
Experimental features	We performed strain-dependent geometry optimizations within the local density approximation of density functional theory (DFT). Dynamical matrices were obtained with the DFT forces and the small displacement method.
Data source location	Korea Advanced Institute of Science and Technology (KAIST), 291 Daehak-ro, Yuseong-gu, Daejeon 305-701, Korea
Data accessibility	All data are presented in this article.
Related research article	H. S. Kim, T. H. Kim, Y.-H. Kim, Odd-even phonon transport effects in strained carbon atomic chains bridging graphene nanoribbon electrodes, Carbon 142 (2019) 107–114.

**Value of the data**

• The data can be references to the atomistic modeling of carbon atomic chains bridging graphene and graphene nanoribbon electrodes with strain effects.
• The data can be references to the quantum phonon transport calculations of carbon atomic chains, graphene nanoribbons, and their heterojunctions.
• The data may be generally relevant for the researchers interested in the quantum transport properties of all-carbon devices.

## 1 Data

**Fig. 1:**
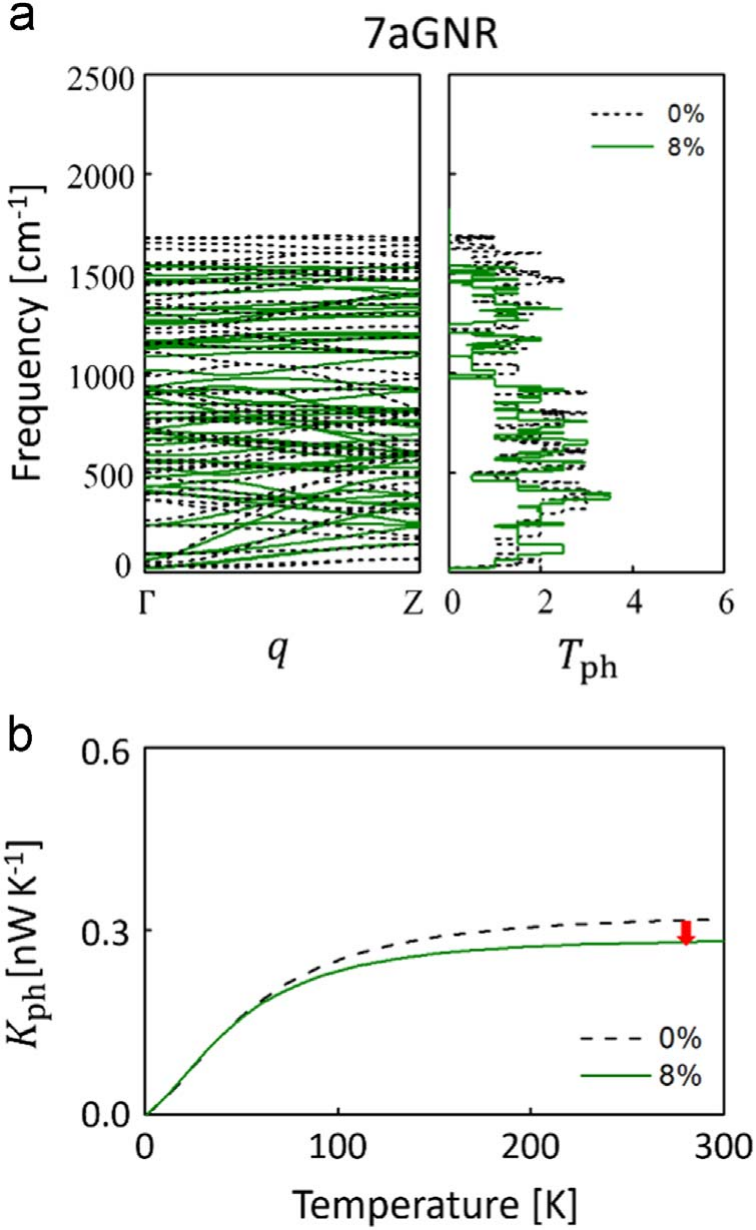
Strain-dependent phonon band structure, phonon transmission (*T*_ph_), and lattice thermal conductance (*K*_ph_) of infinite hydrogen-passivated seven dimer-line armchair graphene nanoribbon (7aGNR). (a) Strain-dependent phonon band structure (left panel) and the corresponding phonon transmission (right panel) of 7aGNR. (b) Strain-dependent lattice thermal conductance of 7aGNR as a function of temperature. Black dashed and green solid lines represent 0% and 8% strain condition, respectively.

**Fig. 2:**
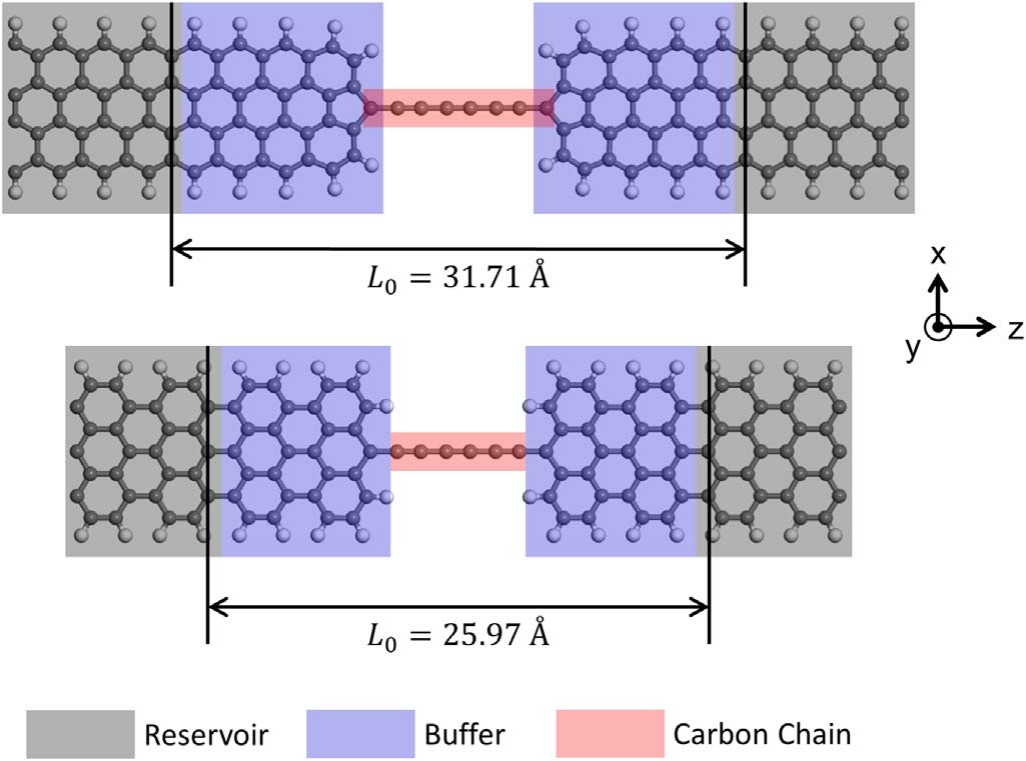
Schematics of the GNR–CC–GNR junction models for the phonon quantum transport calculations. Grey, blue, and red regions are the heat reservoir, buffer, and carbon chain (CC) regions, respectively. We considered two types of GNR types, namely hydrogen-passivated four zigzag-chain zigzag GNR (4zGNR, width *W* = 9.31 Å) and 7aGNR (*W* = 9.27 Å). Six CC (5–10 CCs) and four CC (3–6 CCs) cases are considered for the 4zGNR and 7aGNR electrode cases, respectively. We defined four (two) fixed 4zGNR (7aGNR) unit cells as heat reservoirs. The CCs and their adjacent four (two) 4zGNR (7aGNR) unit cells (buffer) were defined as the phonon scattering region. The distance between the two fixed electrode regions *L*_0_ is given in each case.

**Fig. 3:**
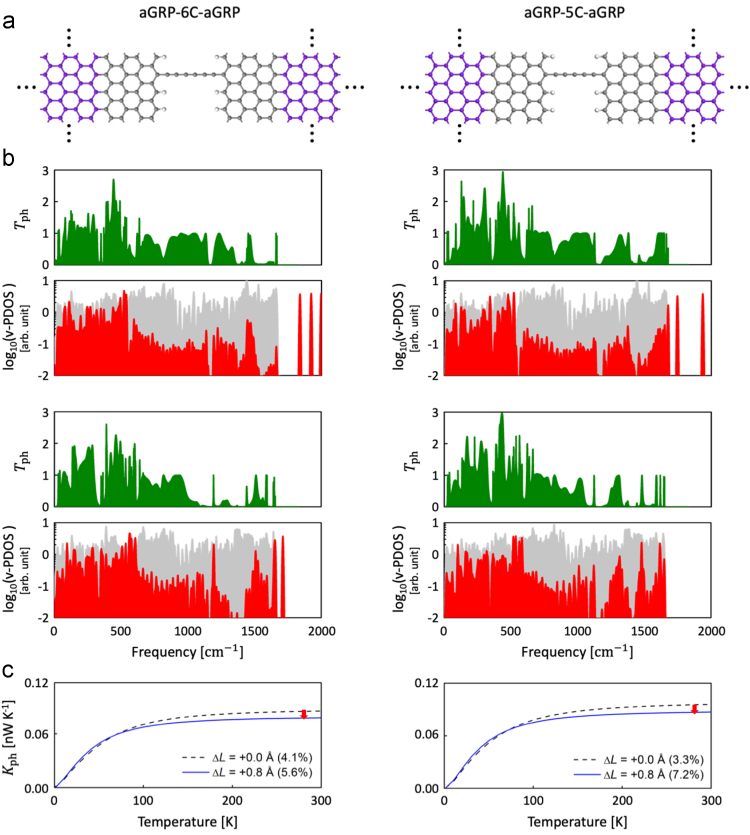
Atomic structures and thermal properties of periodic aGRP electrodes. (a) Schematics of aGRP–6C–aGRP and aGRP–5C–aGRP junction models. (b) Phonon transmissions and vibrational projected density of states (v-PDOS). Green filled lines represent phonon transmissions. Red and grey filled lines represent the v-PDOS of CC and aGRP parts, respectively. (c) Strain-dependent lattice thermal conductances of the two junction models. Black dotted and blue solid lines represent the unstrained (dashed lines) and strained (solid lines) conditions, respectively. Here, *∆L* represents the displacement from the initial junction geometry (*L*_0_ in Fig. 2). Effective strain values for the innermost carbon atoms with reference to the infinite polyyne atomic structure are given together.

**Fig. 4:**
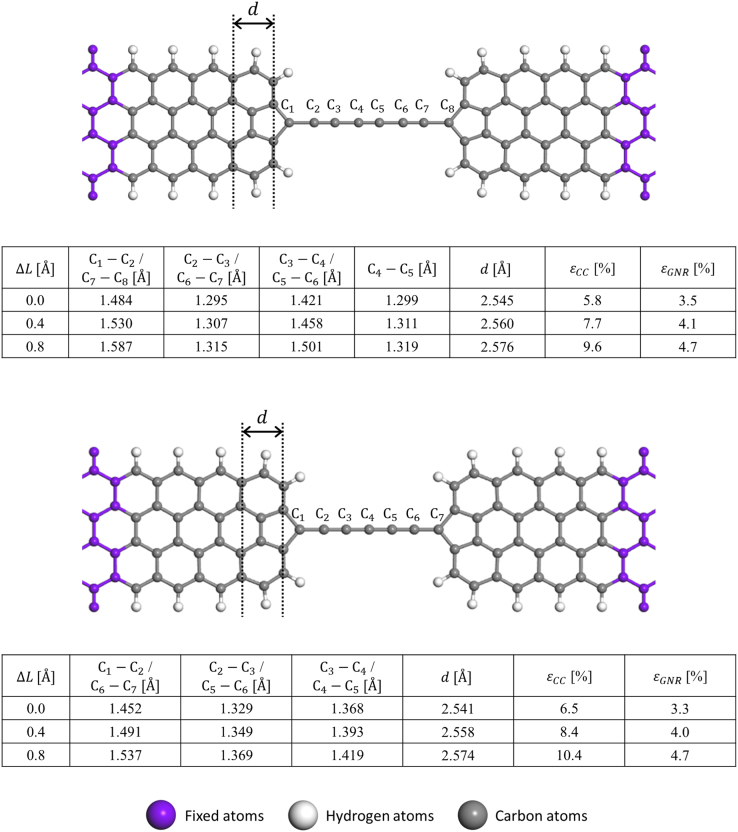
Details of stretching-induced variations in the bond lengths of the 4zGNR–8C–4zGNR (upper panel) and 4zGNR–7C–4zGNR (lower panel) junction models. Here, ∆*L* is the displacement imposed by moving each electrode outward by ∆*L*/2. The length of the innermost 4zGNR unit cell (d) was used to estimate the effective strain on 4zGNRs in the interface region (*ε*_GNR_) compared to the optimized infinite 4zGNR (*d* = 2.46 Å). The effective strain on CC (*ε*_cc_) was defined based on the C_2_–C_3_–C_4_ length and the length of infinite polyyne or twice the cumulene unit cell (2.57 Å). Refer also to the accompanying atomic structure files for details.

**Fig. 5:**
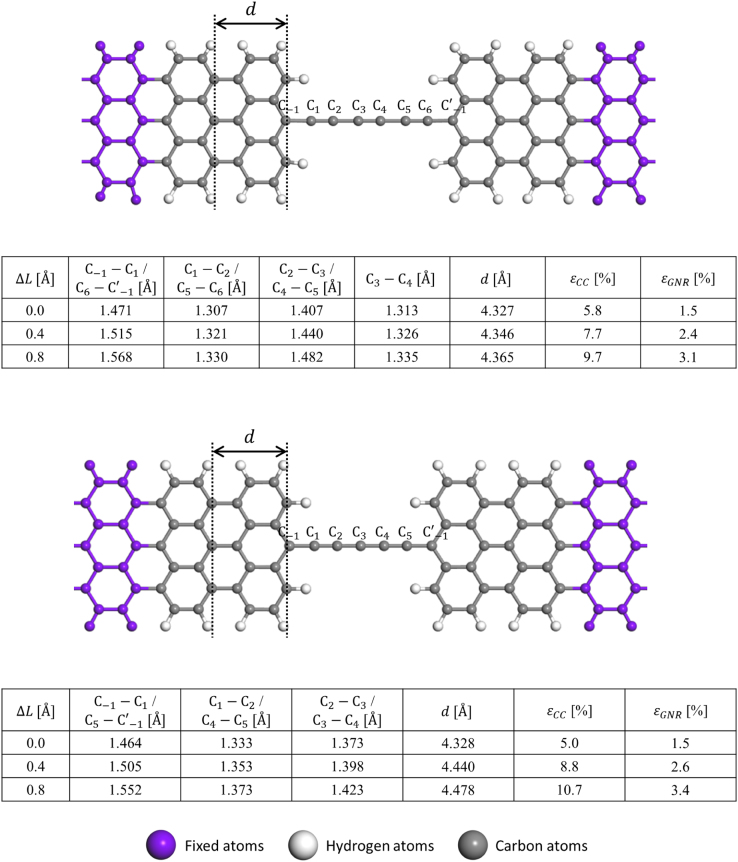
Details of stretching-induced variations in the bond lengths of the 7aGNR–6C–7aGNR (upper panel) and 7aGNR–5C–7aGNR (lower panel) junction models. Note that C_-1_ and C’_-1_ are not counted as the part of CC. Here, *∆L* is the displacement imposed by moving each electrode outward by *∆L*/2. The length of the innermost 7aGNR unit cell (*d*) was used to estimate the effective strain on 7aGNRs in the interface region (*ε*_GNR_) with respect to the optimized infinite 7aGNR (*d* = 4.33 Å). The effective strain on CC (ε_cc_) was defined based on the C_2_–C_3_–C_4_ bond length and the length of the infinite polyyne or twice the cumulene unit cell (2.57 Å).

**Fig. 6:**
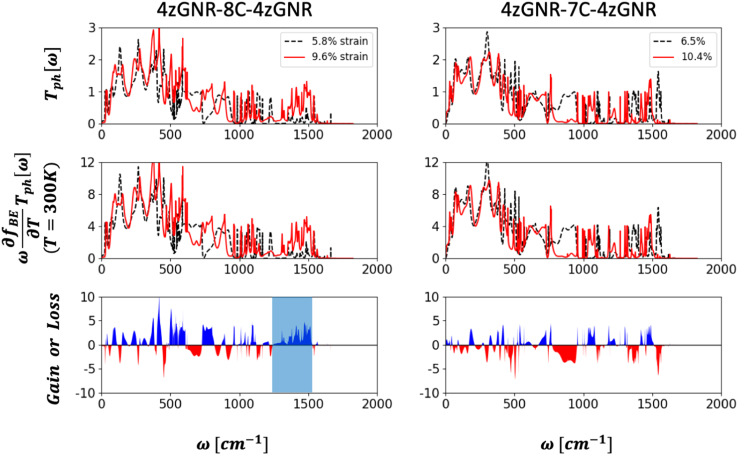
Details of the contributions of *T*_ph_ at all frequency range to *K*_ph_(*T*) in the 4zGNR–8CC/7CC–4zGNR junction models. Top panels show strain-dependent *T*_ph_. Black dashed and red solid lines represent the low (*ε*_cc_ = 5.8% for 8C and 6.5% for 7C) and high strain (*ε*_cc_ = 9.6% for 8C and 10.4% for 7C) conditions, respectively. Middle panels are integrands in the evaluation of *K*_ph_ at *T* = 300 K. Bottom panels show the differences between the integrands of the high- and low-strain conditions. Here, blue (red) filled line represents the gain (loss) of *K*_ph_(*T* = 300 K) at each frequency. The blue shaded region in the bottom left panel indicates the *ω* = 1250–1500 cm^−1^ region in which the gain components are dominant.

In this data article, we present the data on the atomic configurations and corresponding lattice thermal conductances of carbon atomic chains (CCs) bridging graphene nanoribbon (GNR) and graphene (GRP) electrodes [1]. We employed two types of GNRs, namely hydrogen-passivated four zigzag-chain zigzag GNR (4zGNR) and hydrogen-passivated seven dimer-line armchair graphene nanoribbon (7aGNR). In addition, as a counter example, we considered the infinite-width armchair GNR or zigzag-edged graphene (aGRP) electrode case. In Fig. 1, we present the phonon band structure, density of states (DOS), and thermal conductance data obtained for 7aGNR, which can be compared with the 4zGNR data presented in Ref. [1, Fig. 2(c)]. The atomistic models for the 4zGNR–CC–4zGNR and 7aGNR–CC–7aGNR junctions are schematically shown in Fig. 2, and the computational setting for quantum phonon transport calculations is explained in Section 2.2. In Fig. 3, we present the computational data obtained for the aGRP-based junction models. Details of the stretching-induced variations in the CC geometries within the 4zGNR–CC–4zGNR (Fig. 4) and 7aGNR–CC–7aGNR junction models (Fig. 5) are also provided. The atomic structures of the 4zGNR-based junction models (Fig. 5) for the five/six-carbon (5C/6C) chain cases are provided as a zip file in the general xyz format with the file names of “4zGNR-5/6C-4zGNR+{*displacement*}.xyz”. The simulation cell information are provided in the comment lines within the xyz files. In Fig. 6, detailed analyses of phonon transmission spectra are provided for the 4zGNR–based seven-carbon (7C) and eight-carbon (8C) junction models in view of the thermal conductance changes with strain.

## 2 Experimental design, materials, and methods

### 2.1 First‐principles phonon and quantum phonon transport calculation methods

For the junction models described in Fig. 2, atoms inside the scattering region (CC plus buffer GNR or GRP) were relaxed using the local density approximation of density functional theory (DFT) implemented in the SIESTA package [2]. Dynamical matrices were then obtained with the DFT forces and the small displacement method using the Phonopy code [3]. Norm-conserving pseudopotentials and double-zeta-plus-polarization quality atomic orbital basis sets were adopted. The convergence for the total energy was chosen as 10^−6^ eV, and atomic relaxations were performed until the Hellmann–Feynman forces on each atom fall below 10^−3^ eV/Å.

For the computation of ballistic phonon transport properties, we used an in-house code that implements the atomistic matrix Green’s function (MGF) formalism [4], [5], [6], [7] and was developed based on our electronic MGF code [8], [9], [10]. We are concerned with the linear response limit, or when the difference of the electrode 1/2 temperature $T_{1/2}$ is very small, $T_{1}-T_{2}\ll T\equiv \left(T_{1}+T_{2}\right)/2$. Then, after computing the phonon transmission function,

(1) $$T_{\mathrm{ph}}\left(\omega \right)=\mathrm{Tr}\left[\Gamma_{1}\left(\omega \right)G\left(\omega \right)\Gamma_{1}\left(\omega \right)G^{+}\left(\omega \right)\right],$$

where G is the retarded Green׳s function matrix of the channel region and $\Gamma_{1/2}$ is the broadening matrix resulting from the coupling of the channel with the electrode 1/2, we calculated the lattice thermal conductance according to

(2) $$K_{\mathrm{ph}}\left(\omega \right)=\int_{0}^{\infty }\frac{d\omega }{2\pi }\hbar \omega T\left(\omega \right)\frac{\partial n}{\partial T},$$

where $n={\left[\exp\left(\hbar \omega /k_{B}T\right)-1\right]}^{-1}$ is the Bose–Einstein distribution function. To analyze the dominant phonon transmission eigenmodes, we visualized their phonon local PDOS in a similar spirit of visualizing electronic eigenstates of the molecular projected Hamiltonian in the electron quantum transport calculations [11], [12].

### 2.2 Construction of GNR–CC–GNR and GRP–CC–GRP junction models for quantum phonon transport calculations

The geometries and quantum phonon transport properties of the GNR–CC–GNR and GRP–CC–GRP junction models were obtained following the procedure established earlier for the study of quantum electron transport properties in molecular [10], [13] and semiconductor junctions [14]. For example, searching for the optimal gap distance for the 4zGNR–8C–4zGNR junction case by carrying out energy minimizations, we obtained the innermost C–C and C≡C bond distances of 1.421 Å and 1.299 Å, respectively (the bond-length alternation of 0.123 Å). On the other hand, for the 4zGNR–7C–4zGNR junction model, the internal C=C distance was 1.368 Å.

Expecting the CC parts will adopt similar configurations within the zGNR and aGNR electrodes, junction models based on 7aGNR electrodes were initially prepared by extracting the inner CC parts (excluding the carbon atom at the pentagonal tip) in the 4zGNR–CC–4zGNR junction models and replacing the 4zGNRs by 7aGNRs. The scattering-region atoms were energy minimized once again. For the aGRP cases, we repeated a similar procedure to expedite model preparations. After obtaining the optimal geometries of the GNR–CC–GNR and GRP–CC–GRP junction models, we stretched junction model by moving GNR or GRP electrodes outward by *ΔL* = 0.4 Å and 0.8 Å and repeated energy minimization procedures.
