# Supplementary material for: Data for quantum phonon transport in strained carbon atomic chains bridging graphene and graphene nanoribbon electrodes
Source: Data Brief. 2018 Nov 15;21:2421–9. doi: 10.1016/j.dib.2018.11.049 (PMC6282110; doi:10.1016/j.dib.2018.11.049)
Supplement: Supplementary file 2 — Supplementary material [file mmc2.zip › CC+GNR_Carbon-Rev_DiB.docx]

DATA IN BRIEF TEMPLATE

**Meta-Data (Mandatory information required for the transfer of your article to Data in Brief – will not be typeset)**

| ***Title:** | Data and supplemental information for odd-even phonon transport effects in strained carbon atomic chains bridging graphene nanoribbon electrodes |
| --- | --- |
| ***Authors:** | *Hu Sung Kim ^a^ Tae Hyung Kim,^a^ and Yong-Hoon Kim^a,*^* |
| ***Affiliations:** | *^a^School of Electrical Engineering and Graduate School of EEWS, Korea Advanced Institute of Science and Technology, 291 Daehak-ro, Yuseong-gu, Daejeon 305-701, Korea* |
| ***Contact email:** | [*y.h.kim@kaist.ac.kr*](mailto:y.h.kim@kaist.ac.kr) |
| *****Co-authors**: | Hu Sung Kim ([husungkim@kaist.ac.kr](mailto:husungkim@kaist.ac.kr))  Tae Hyung Kim ([ramanujankim@kaist.ac.kr](mailto:ramanujankim@kaist.ac.kr)) |
| ***CATEGORY:** | *Nanotechnology* |

**Data Article**

**Title**: Data and supplemental information for odd-even phonon transport effects in strained carbon atomic chains bridging graphene nanoribbon electrodes

**Authors**: Hu Sung Kim,^a^ Tae Hyung Kim,^a^ and Yong-Hoon Kim^a,*^

**Affiliations**: ^a^School of Electrical Engineering and Graduate School of EEWS, Korea Advanced Institute of Science and Technology, 291 Daehak-ro, Yuseong-gu, Daejeon 305-701, Korea

**Contact email**: y.h.kim@kaist.ac.kr

**Abstract**

The data presented in this paper is the supplementary data of “odd-even phonon transport effects in strained carbon atomic chains bridging graphene nanoribbon electrodes”. To provide the information that can be utilized in reproducing our data, we provide the fully optimized graphene nanoribbon (GNR)-carbon chain (CC)-GNR junction atomic configurations at different strain values, the computational setting for quantum phonon transport calculations, and several computational data that were not included in the main manuscript.

**Specifications Table**

| Subject area | Physics |
| --- | --- |
| More specific subject area | Computational condensed matter |
| Type of data | Table, figure, file |
| How data was acquired | Computational modeling and simulations |
| Data format | Analyzed |
| Experimental factors | - |
| Experimental features | We carried out strain-dependent geometry optimizations within the local density approximation of density functional theory (DFT). Dynamical matrices were obtained with the DFT forces and the small displacement method. |
| Data source location | Korea Advanced Institute of Science and Technology,  291 Daehak-ro, Yuseong-gu, Daejeon 305-701, Korea |
| Data accessibility | All data are presented in this article. |
| Related research article | H. S. Kim, T. H. Kim, Y.-H. Kim, Odd-even phonon transport effects in strained carbon atomic chains bridging graphene nanoribbon electrodes, Carbon (2018). In press. |

**Value of the Data**

- The data show the specific geometries and the corresponding lattice thermal conductance of carbon atomic chains bridging graphene nanoribbon electrodes with strain effects.
- The data highlight the difference between even- and odd-numbered carbon atomic chains in terms of thermal properties.
- The data indicate that carbon atomic chains show different response to tensile strain.
- The data may be relevant for other researchers interested in thermal properties of carbon-based devices.

**1. Data**

In this report, we present data on the specific atomic configurations and the corresponding lattice thermal conductance of carbon atomic chains (CCs) bridging graphene nanoribbon (GNR) electrodes [1]. We considered two types of GNRs, namely hydrogen-passivated four zigzag-chain zigzag GNR (4zGNR) and hydrogen-passivated seven dimer-line armchair graphene nanoribbon (7aGNR). In addition, as a counter example, we also considered the infinite-width armchair GNR or zigzag-edged graphene (aGRP) electrode case. In Figs. 1 and 3, we present the computational data obtained for the 7aGNR and aGRP-6C-aGRP junction models. The computational setting for quantum phonon transport calculations is explained in Fig. 2. Details of the stretching-induced variations in the CC geometries of the 7aGNR-CC-7aGNR junction models (Fig. 4) and 4zGNR-CCs-4zGNR junction models (Fig. 5) are also provided.


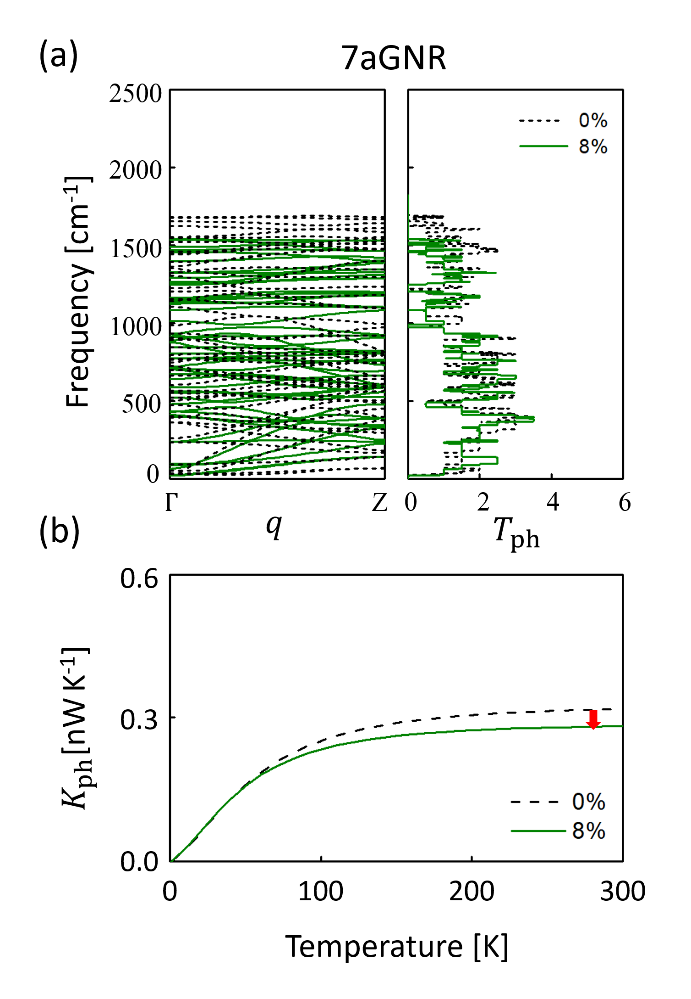


**Fig. 1.** Strain-dependent phonon band structure, phonon transmission ($T_{\mathrm{ph}}$), and lattice thermal conductance ($K_{\mathrm{ph}}$) of infinite hydrogen-passivated seven dimer-line armchair graphene nanoribbon (7aGNR). (a) Strain-dependent phonon band structure (left panels) and the corresponding phonon transmission (right panels) of 7aGNR. (b) Strain-dependent lattice thermal conductance of 7aGNR as a function of temperature. Black dashed and green solid lines represent 0 and 8% strain condition, respectively.


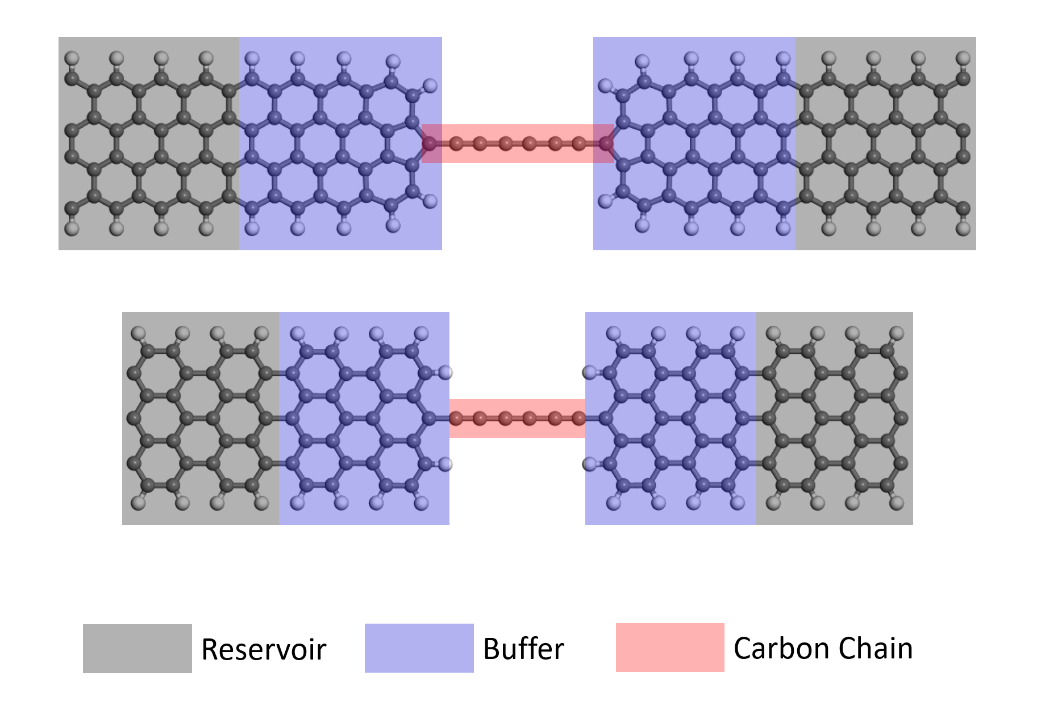


**Fig. 2**. Details of the construction of GNR-CC-GNR junction models for phonon quantum transport calculations. Grey, blue, and red rectangles are heat reservoir, buffer, and carbon chain (CC) regions, respectively. We considered two types of GNRs, namely hydrogen-passivated four zigzag-chain zigzag GNR (4zGNR, width *W* = 9.31 Å) and 7aGNR (*W* = 9.27 Å). Six and four CC cases are considered in each GNR case (5—10 CCs for the 4zGNR case and 3—6 CCs for the 7aGNR case). We defined four (two) fixed 4zGNR (7aGNR) unit cells as heat reservoirs. The CCs and their adjacent four (two) 4zGNR (7aGNR) unit cells (buffer) were defined as the phonon scattering region. The atoms inside the scattering region were relaxed using density functional theory to find their optimal positions. Here, the convergence for the total energy was chosen as 10^−6^ eV, and atomic relaxations were performed until the Hellmann-Feynman forces on each atom fall below 10^−3^ eV/Å. After finding the optimal geometry of the 4zGNR-CC-4zGNR junctions, we stretched each junction model along the chain direction by moving each GNR electrode outward by 0.4 Å (total of 0.8 Å extension). At the optimal gap distance, the bond-length alternation obtained for the innermost carbon atoms was 0.123 Å for the 4zGNR-8C-4zGNR junction model (C – C and C ≡ C bond distances were 1.421 Å and 1.299 Å, respectively). For the 4zGNR-7C-4zGNR junction model, the internal C=C distance was 1.368 Å. Expecting the CC parts will adopt similar configurations within the zGNR and aGNR electrodes, junction models with 7aGNR electrodes were initially prepared by extracting the inner CC parts (excluding the carbon atom at the pentagonal tip) in the 4zGNR-CC-4zGNR junction models and replacing the 4zGNRs by 7aGNRs. After that, the atoms belong to the scattering region were relaxed again and the tensile strain was applied using the same method as in the 4zGNR-CC-4zGNR junction case (0.8 Å displacements).


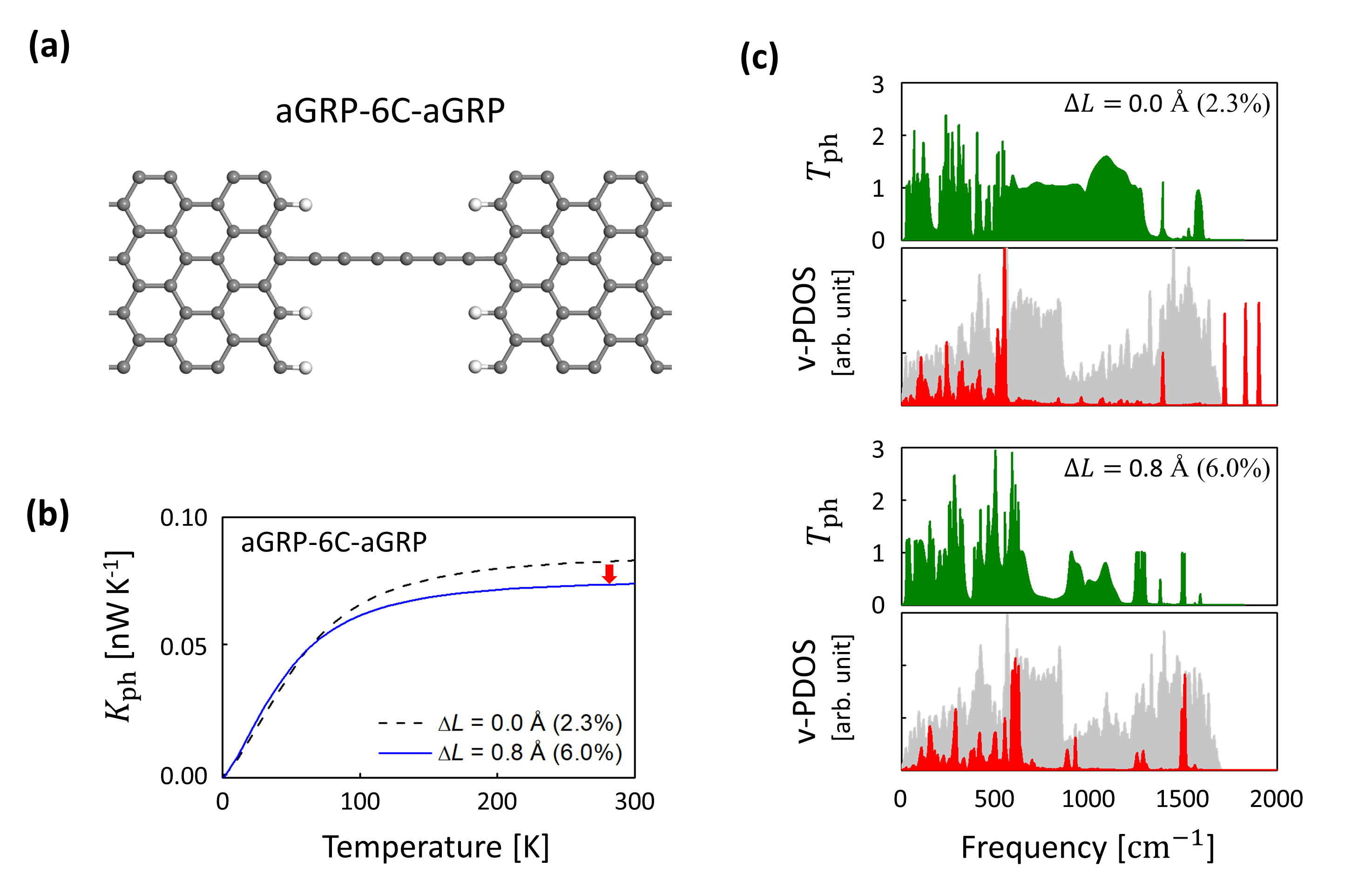


**Fig. 3.** Atomic structures and thermal properties of periodic graphene electrodes with eight dimer-line armchair unit (aGRP). (a) Schematics of aGRP-6C-aGRP junction contacts. (b) Strain-dependent lattice thermal conductances of the aGRP-6C-aGRP junction models. Black dotted and blue solid lines represent the unstrained (dashed lines) and strained (solid lines) conditions, respectively. Here, *∆L* is the displacement from the initial geometry. Effective strain values for the innermost carbon atoms in reference to the infinite polyyne atomic structure are indicated together. (c) Phonon transmissions and vibrational projected density of states (v-PDOS). Green filled lines represent phonon transmission. Red and grey filled lines represent the v-PDOS of CC and GRP parts, respectively.


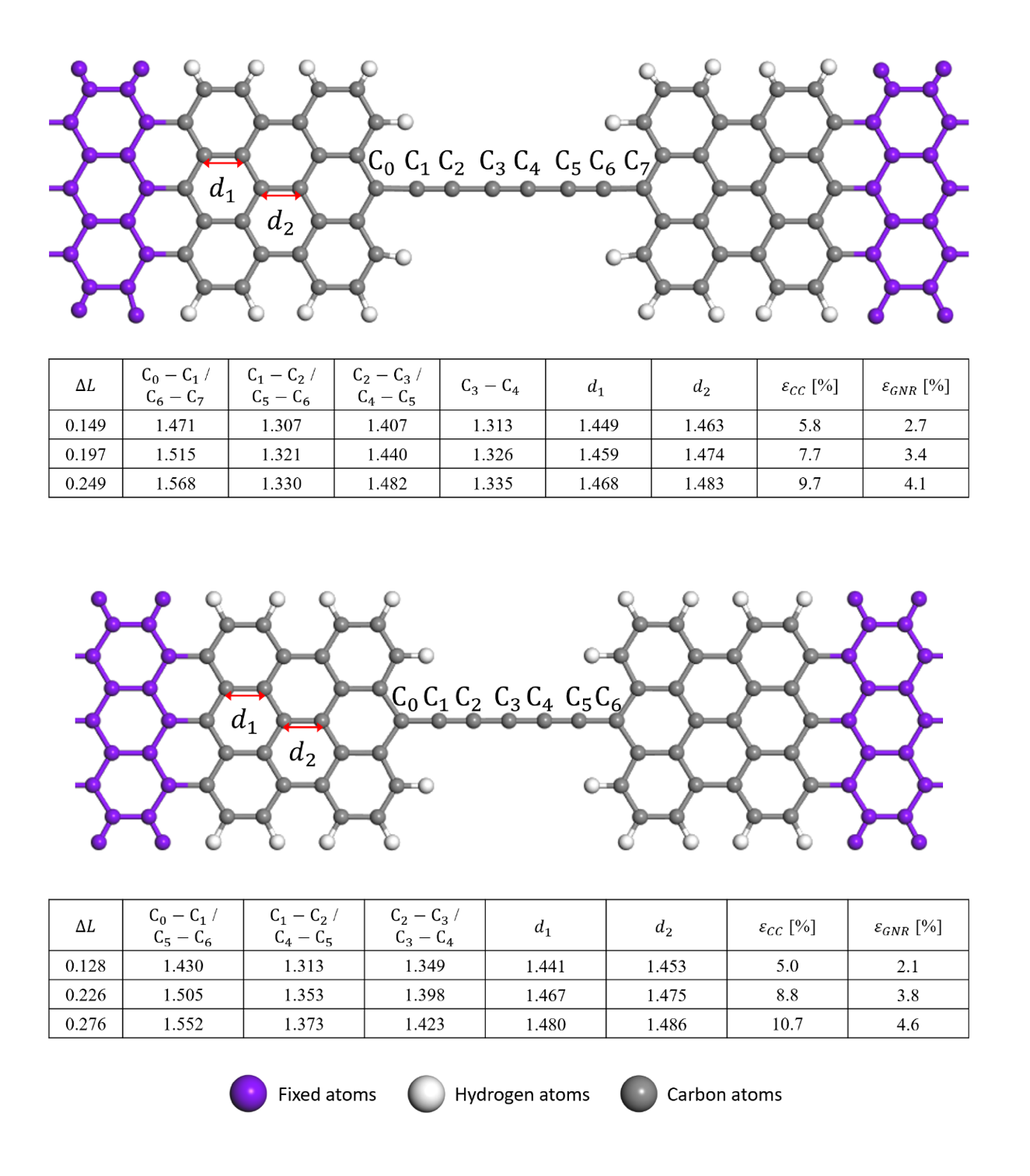


**Fig. 4.** Details of stretching-induced variations in the bond lengths of the 7aGNR-6C-7aGNR (upper panel) and 7aGNR-5C-7aGNR (lower panel) junction models. Note that $C_{0}$ and $C_{7}$ ($C_{6}$) are not counted as the parts of the 6C (5C). Two representative C-C bond lengths inside 7aGNR, $d_{1}$ and $d_{2}$, were measured to estimate the effective strain on GNRs in the interface region ($\varepsilon_{GNR}$) with respect to the energy-optimized infinite 7aGNR ($d$ = 1.43 Å). The effective strain on CC ($\varepsilon_{CC}$) was defined based on the $C_{2}-C_{3}-C_{4}$ length and the length of infinite polyyne unit cell (2.57 Å) for the 6C case or infinite cumulene 2-atom unit cell (2.57 Å) for the 5C case. Here, the corresponding amount of deformation (*∆L*) was defined as $\varepsilon_{CC}\times2.57Å$.


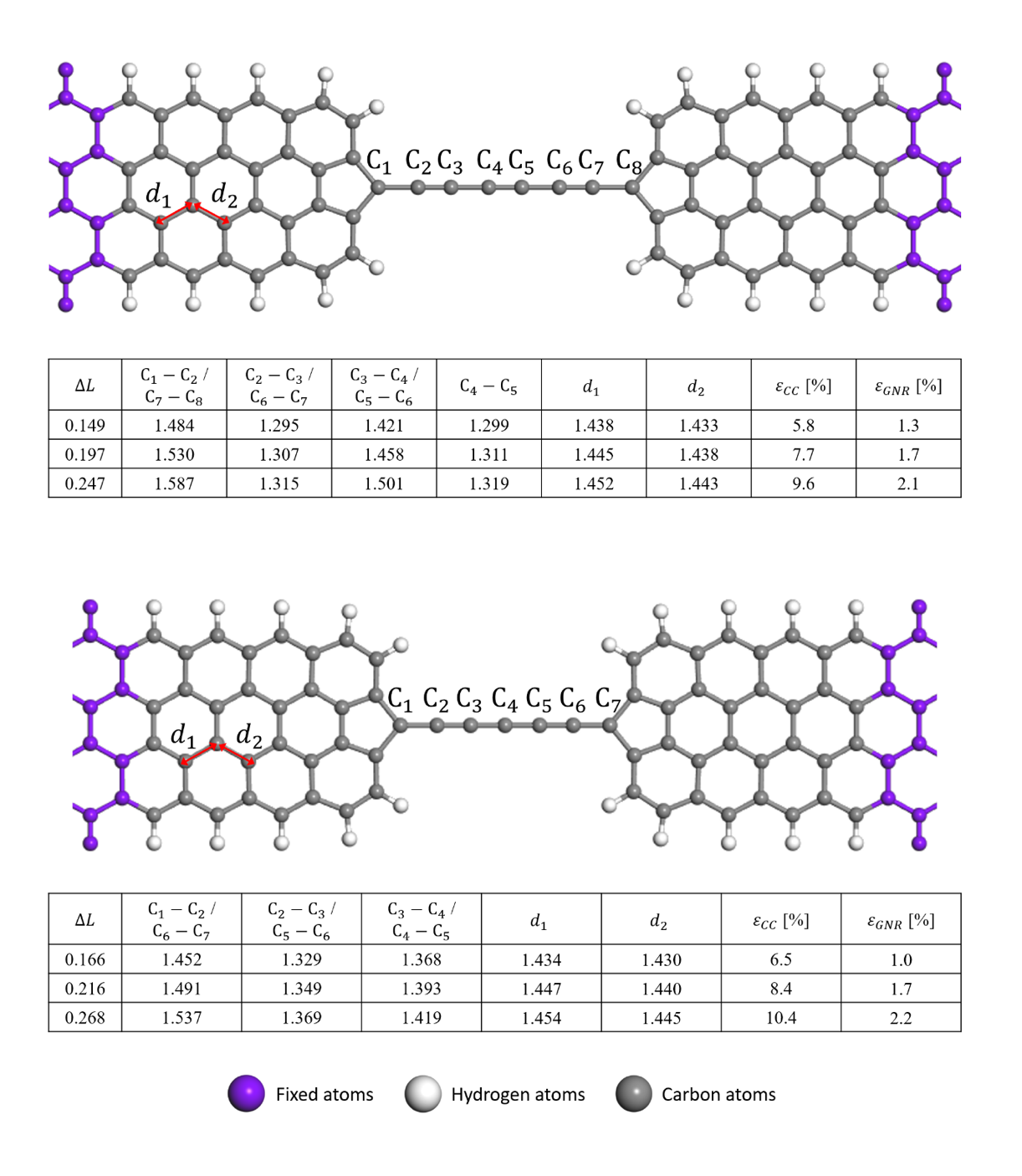


**Fig. 5.** Details of stretching-induced variations in the bond lengths of the 4zGNR-8C-4zGNR (upper panel) and 4zGNR-7C-4zGNR (lowerpanel) junction models. Two representative C-C bond length inside 4zGNR, $d_{1}$ and $d_{2}$ were measured to estimate the effective strain on GNRs in the interface region ($\varepsilon_{GNR}$) with respect to the energy-optimized infinite 7zGNR ($d$ = 1.42 Å). The effective strain on CC $\varepsilon_{CC}$ was defined based on the $C_{3}-C_{4}-C_{5}$ bond length and the length of the infinite polyyne unit cell (2.57 Å) for the 8C case or the infinite cumulene 2-atom unit cell (2.57 Å) for the 7C case. Here, the corresponding amount of deformation (∆L) was defined as $\varepsilon_{CC}\times2.57Å$. Also, refer to the accompanying atomic structure files for details.


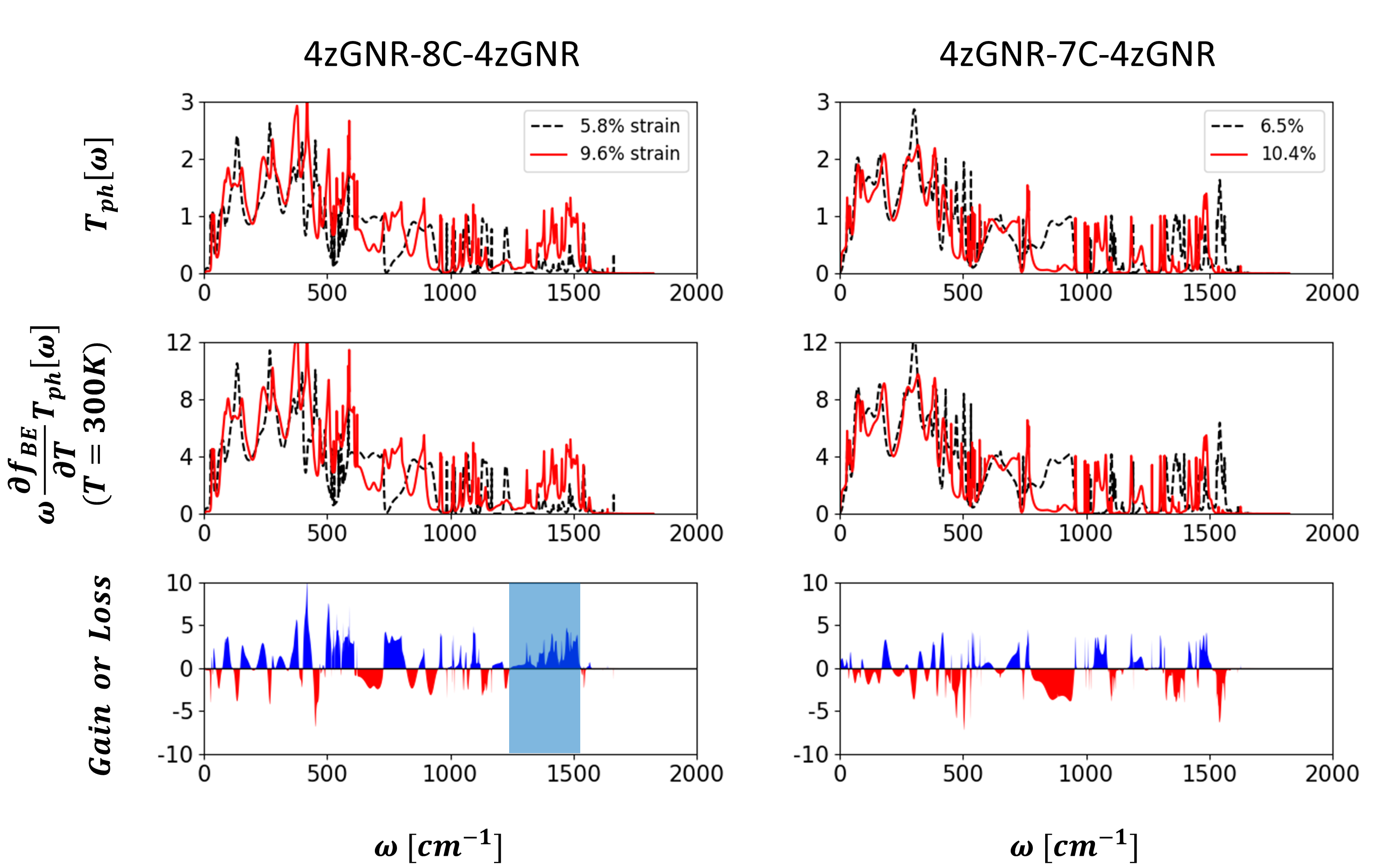


**Fig. 6.** Details of the contribution of all frequency range on the $K_{\mathrm{ph}}\left( T \right)$ of 4zGNR-CCs-4zGNR junction models. Top panels represent strain-dependent $T_{\mathrm{ph}}$. Black dashed and red solid lines represent the low ($\varepsilon_{CC}$ = 5.8% for 8C and 6.5% for 7C) and high strain ($\varepsilon_{CC}$ = 9.6% for 8C and 10.4% for 7C) conditions, respectively. Middle panels are integrand for evaluation of $K_{\mathrm{ph}}\left( T \right)$. Bottom panels show the difference between the integrand of the high and low strain conditions. Here, blue (red) filled line represents gain (loss) of $K_{\mathrm{ph}}\left( T \right)$ at certain frequency. The transparent blue rectangle in left bottom panel represents ω = 1250 ~ 1500 cm^-1^ frequency region in which the gain components are dominant.

**2. Experimental Design, Materials, and Methods**

*2.1 Density-functional theory phonon calculations*

We carried out strain-dependent geometry optimizations within the local density approximation of density functional theory (DFT) implemented in the SIESTA package.[2] Dynamical matrices were obtained with the DFT forces and the small displacement method using the Phonopy code.[3] Norm-conserving pseudopotentials and double-zeta-plus-polarization quality atomic orbital basis sets were adopted. The convergence criterion for atomic forces was set to 10^−3^ eV/Å.

*2.2 Quantum phonon transport calculations*

For the computation of ballistic phonon transport properties, we used an in-house code that implements the atomistic matrix Green’s function (MGF) formalism[4-7] and was developed based on our electronic MGF code.[8-10] We are concerned with the linear response limit, or when the difference of the electrode 1/2 temperature $T_{1/2}$ is very small, $T_{1}-T_{2}\ll T\equiv\left( T_{1}+T_{2} \right)/2$. Then, after computing the phonon transmission function,

$$T_{\mathrm{ph}}(\omega)=Tr\left[ \boldsymbol{\Gamma}_{1}(\omega)\mathbf{G}(\omega)\boldsymbol{\Gamma}_{2}(\omega)\mathbf{G}^{+}(\omega) \right]$$

where $\mathbf{G}$ is the retarded Green`s function matrix of the channel region and $\boldsymbol{\Gamma}_{1/2}$ is the broadening matrix resulting from the coupling of the channel with the electrode 1/2, we calculated the lattice thermal conductance according to

$$K_{\mathrm{ph}}\left( T \right)=\int_{0}^{\infty} \frac{d\omega}{2\pi}\hbar\omega T(\omega)\frac{\partial n}{\partial T}$$

where $n=\left[ \exp(\hbar\omega/k_{B}T)-1 \right]^{-1}$ is the Bose-Einstein distribution function.

*2.3 Construction of GNR-CC-GNR junction models*

We considered two types of GNRs, namely 4zGNR (width *W* = 9.31 Å) and 7aGNR (*W* = 9.27 Å). Six and four CC cases are considered in each GNR case (5—10 CCs for the 4zGNR case and 3—6 CCs for the 7aGNR case). We defined four (two) fixed 4zGNR (7aGNR) unit cells as heat reservoirs. The CCs and their adjacent four (two) 4zGNR (7aGNR) unit cell buffers were defined as the phonon scattering region. The atoms inside the scattering region were relaxed using DFT to find their optimal positions. Here, the convergence criterion for the total energy was chosen as 10^−6^ eV, and atomic relaxations were performed until the Hellmann-Feynman forces on each atom fall below 10^−3^ eV/Å. After finding the optimal geometry of the 4zGNR-CC-4zGNR junctions, we stretched each junction model along the chain direction by moving each GNR electrode outward by 0.4 Å (total of 0.8 Å extension). At the optimal gap distance, the bond-length alternation (BLA) obtained for the innermost carbon atoms was 0.123 Å for the 4zGNR-8C-4zGNR junction model (C – C and C ≡ C bond distances were 1.421 Å and 1.299 Å, respectively). For the 4zGNR-7C-4zGNR junction model, the internal C=C distance was 1.368 Å. Expecting the CC parts will adopt similar configurations within the zGNR and aGNR electrodes, junction models with 7aGNR electrodes were initially prepared by extracting the inner CC parts (excluding the carbon atom at the pentagonal tip) in the 4zGNR-CC-4zGNR junction models and replacing the 4zGNRs by 7aGNRs. After that, the atoms belong to the scattering region were relaxed again and the tensile strain was applied using the same method as in the 4zGNR-CC-4zGNR junction case (0.8 Å displacements).

**3. Note on the data files**

The atomic structures of the 4zGNR junction models (Fig. 5) for the 5C and 6C cases are provided as a zip file in the general xyz format. The simulation cells are provided in the comment lines within the xyz files. The structure files are named “4zGNR-5/6C-4zGNR_+{*displacement*}.xyz”.

**Acknowledgments**

This work was supported by the Nano-Material Technology Development Program (Nos. 2016M3A7B4024133 and 2016M3A7B4909944), Basic Research Program (No. 2017R1A2B3009872), Global Frontier Program (No. 2013M3A6B1078881), and Basic Research Lab Program (No. 2016M3A7B4909944) of the National Research Foundation funded by the Ministry of Science and ICT of Korea.

**Appendix A. Supplementary material**

Supplementary data associated with this article can be found in the online version at http://dx.doi. org/ … .

**References**

[1] H.S. Kim, T.H. Kim, Y.-H. Kim, Odd-even phonon transport effects in strained carbon atomic chains bridging graphene nanoribbon electrodes, Carbon (2018). In press.

[2] J.M. Soler, E. Artacho, J.D. Gale, A. Garcia, J. Junquera, P. Ordejon, D. Sanchez-Portal, The SIESTA method for ab initio order-N materials simulation, J. Phys-Condens. Mat. 14 (2002) 2745-2779.

[3] A. Togo, L. Chaput, I. Tanaka, G. Hug, First-principles phonon calculations of thermal expansion in Ti3SiC_2_,Ti3AlC_2_, and Ti3GeC_2_, Phys. Rev. B 81 (2010) 174301.

[4] N. Mingo, L. Yang, Phonon transport in nanowires coated with an amorphous material: An atomistic Green’s function approach, Phys. Rev. B 68 (2003) 245406.

[5] T. Yamamoto, K. Watanabe, Nonequilibrium Green's function approach to phonon transport in defective carbon nanotubes, Phys. Rev. Lett. 96 (2006) 255503.

[6] J.-S. Wang, J. Wang, N. Zeng, Nonequilibrium Green’s function approach to mesoscopic thermal transport, Phys. Rev. B 74 (2006) 033408.

[7] B.K. Nikolic, K.K. Saha, T. Markussen, K.S. Thygesen, First-principles quantum transport modeling of thermoelectricity in single-molecule nanojunctions with graphene nanoribbon electrodes, J. Comput. Electron. 11 (2012) 78-92.

[8] Y.-H. Kim, S.S. Jang, Y.H. Jang, W.A. Goddard III, First-principles study of the switching mechanism of [2]catenane molecular electronic devices, Phys. Rev. Lett. 94 (2005) 156801.

[9] Y.-H. Kim, J. Tahir-Kheli, P.A. Schultz, W.A. Goddard III, First-principles approach to the charge-transport characteristics of monolayer molecular-electronics devices: Application to hexanedithiolate devices, Phys. Rev. B 73 (2006) 235419.

[10] Y.-H. Kim, Toward Numerically Accurate First-Principles Calculations of Nano-Device Charge Transport Characteristics: The Case of Alkane Single-Molecule Junctions, J. Kor. Phys. Soc. 52 (2008) 1181-1186.
